# ﻿Multi-origin analysis of the traditional Chinese medicine Leiwan

**DOI:** 10.3897/mycokeys.125.164001

**Published:** 2025-11-26

**Authors:** Wen-Hao Zhang, Guo-Li Zhang, Han-Chi Lei, Chang Xu, Jia Li, Xing Xia, Shuai Jiang, Na Li, Li-Ping Tang

**Affiliations:** 1 School of Pharmaceutical Sciences and Yunnan Key Laboratory of Pharmacology for Natural Products, Kunming Medical University, Kunming, 650500, China; 2 Yunnan College of Modern Biomedical Industry, Kunming Medical University, Kunming 650500, China; 3 Chongqing University of Chinese Medicine, Chongqing, 408435, China; 4 Chongqing Medical University, Chongqing, 400010, China

**Keywords:** Medicinal fungi, *
Moniliophthora
*, phylogenetics, taxonomy

## Abstract

This study investigates the taxonomic identity of Leiwan (雷丸), a traditional medicinal fungus used in Chinese herbal medicine. Over the past two decades, 21 specimens were collected from herbal markets and through extensive field surveys across multiple provinces in China. Both morphological and phylogenetic analyses reveal that Leiwan originates from multi-evolutionary lineages, represented by at least three distinct species: *Gerronema brunneosquamulosum* (雷丸老伞), *G. sinense***sp. nov.** (中华老伞) and *Moniliophthora sclerotium***sp. nov.** (菌核丛梗霉皮伞). We confirm that *G. brunneosquamulosum* is a valid name for the traditional Chinese medicine Leiwan and propose *G. lapidescens* as its synonym. Furthermore, *M. sclerotium* is the first report of a sclerotium-forming species within this genus, marking a significant expansion of its known ecological and morphological diversity. This study provides a taxonomic framework for Leiwan, establishing a scientific basis for future mycological and pharmacological research.

## ﻿Introduction

Leiwan, also called Zhuling, Zhulingzhi, Mulianzi and Leigongwan, was first recorded in the *Shennong BenCao Jing* as a traditional Chinese medicinal fungus (Wang et al. 2008). With over 2,000 years of medicinal history, it has been included in the “Pharmacopoeia of the People’s Republic of China” since 1963, where it is used as dried sclerotia (National Pharmacopoeia Commission 1963). Historical texts note its association with the stomach and large intestine meridians and it is valued for anthelmintic and digestive therapeutic effects (Chen and Bao 2012). It has been used to treat tapeworm (Jin 1990), hookworm (Li and Chen 1957), and roundworm infections (Hou and Peng 1952), and to relieve abdominal pain caused by parasites and paediatric indigestion (Lian 1989; Guo 2014). As a widely used vermifuge, contemporary research has revealed that it contains numerous bioactive compounds, including omphalin, ergosterol, polysaccharides and the protein pPeOp, which exhibit insecticidal (Zhao et al. 1998), anti-inflammatory (Wang and Zhu 1989; Zhu et al. 2016), antioxidative (Li et al. 2020), antitumour (Yao and Ma 1979; Liang 2012; Chen et al. 2018) and hypoglycaemic properties (Zhang et al. 2006). Owing to its safe anthelmintic effects and potential antitumour applications, Leiwan is regarded as a promising medicinal fungus in China (Huang 2005), though its taxonomic origin has long been controversial.

The taxonomic history of Leiwan is complex. In 1856, the Russian missionary Horaninow first described it as *Mylitta lapidescens* Horan., based on sclerotium specimens from Beijing, China — a name that was subsequently used in some references (Preparation Group of National Compendium of Chinese Herbal Medicine 1975). In 1891, German mycologists F. Cohn and J. Schroeter cultivated sclerotia of Leiwan to obtain fruiting bodies and reclassified it as *Omphalia lapidescens* (Horan.) F. Cohn & J. Schroeter (Liu and Lin 2006). This latter name has been widely adopted in literature, including its designation in the Pharmacopoeia of the People’s Republic of China as the official origin of Leiwan (National Pharmacopoeia Commission 1963). However, some researchers have used *Polyporus mylittae* Cooke & Massee, a fungus originally described from Australia, as the source of Leiwan (Zhao et al. 1998; Yu 2002). This taxon was later treated as a synonym of *Laccocephalum mylittae* (Cooke & Massee) Núñez & Ryvarden (Dai and Yang 2008).

Previously, the identification of Leiwan relied on morphology until Zhang et al. (2024b) used ITS sequence analysis and morphological characteristics to reclassify it into the genus *Gerronema* Singer, renaming it *G. lapidescens* (Horan.) Ming Zhang & W.X. Zhang, thereby correcting Leiwan’s generic placement. Notably, subsequent phylogenetic analyses (Zhang et al. 2025) revealed that *G. brunneosquamulosum* Q. Na & Y.P. Ge, a species newly described from Zhejiang Province, China (Na et al. 2024) and characterised by a brownish pileus with radial striations, was found nesting within the *G. lapidescens* clade, suggesting close affinity between the two.

To clarify the taxonomic origin of Leiwan, we conducted an investigation over two decades, collecting 21 samples from traditional Chinese medicine markets and natural habitats across China. Integrated morphological observations and phylogenetic analyses revealed that commercial Leiwan comprises at least three distinct taxa: *G. brunneosquamulosum* and two newly-described species, *G. sinense* sp. nov. and *Moniliophthora sclerotium* sp. nov. Our study also confirms that *G. lapidescens* should be treated as a synonym of *G. brunneosquamulosum*.

The genus *Gerronema*, established by Singer, includes small wood-inhabiting agarics and is currently assigned to the family Porotheleaceae Murrill (Singer 1951; Na et al. 2024; Zhang et al. 2025). In contrast, the genus *Moniliophthora* H.C. Evans, Stalpers, Samson & Benny, belonging to the family Marasmiaceae Roze ex Kühner, was first proposed in 1978 to accommodate the anamorphic pathogen *M. roreri*, a major causal agent of frosty pod rot in cocoa (Evans et al. 1978). Species of *Moniliophthora* are hemibiotrophic pathogens of considerable ecological and economic importance (Haqnawaz et al. 2024).

A key finding of this study is the discovery of a novel species, *Moniliophthora sclerotium*, which represents the first report of a sclerotium-forming species within this genus. Although complete morphological data are presently unavailable, its distinct phylogenetic position strongly supports its recognition as a new species. This discovery not only expands the known taxonomic sources of Leiwan, but also offers new insights into the ecological adaptation and evolutionary history of *Moniliophthora*.

## ﻿Materials and methods

In this study, we examined 21 specimens of Leiwan, collected from Anhui, Chongqing, Guangxi, Guizhou, Hainan, Sichuan and Yunnan. Specimen vouchers are deposited in the Mycological Herbarium of Kunming Medical University (MHKMU) and the Chongqing Institute of Medicinal Plant Cultivation.

Macroscopic descriptions were based on field notes and digital photos of the basidiomata; colour designations follow Kornerup and Wanscher (1981). Microscopic observations were made from dried material rehydrated in 5% potassium hydroxide (KOH) and stained with 1% Congo Red solution (w/v), using a Leica DM2500 light microscope. Melzer’s reagent was used to test the amyloidity of basidiospores. In the basidiospore description, [n/m/p] indicates “n” basidiospores measured from “m” basidioma of “p” collections. Measurement ranges are expressed as “(a) b–c (d)”, where “b–c” covers at least 90% of the data and “a” and “d” are extremes. Basidiospore shapes were classified according to the length-to-width ratio “Q”, using the following Q ranges in side view, following Bas (1969): globose (1.0–1.05), subglobose (1.05–1.15), broadly ellipsoid (1.15–1.3), ellipsoid (1.3–1.6), and elongate (1.6–2.0). The mean Q value “Q_m_” per collection is given as mean ± sample standard deviation.

### ﻿DNA extraction, PCR amplification and sequencing

DNA extraction, PCR amplification and sequencing followed Tang et al. (2015). Approximately 10–20 mg of dried tissue was used for genomic DNA extraction. PCR amplification of the ITS region (nuclear ribosomal internal transcribed spacer) and nrLSU (nuclear large subunit ribosomal) employed primer pairs ITS5/ITS4 (White et al. 1990) and LR0R/LR5 (Vilgalys and Hester 1990), respectively.

PCR amplification was performed in a 25 μl reaction volume containing 2.5 μl of 10 × amplification buffer (with MgCl_2_), 0.5 μl dNTPs (200 μM), 0.2 μl Taq DNA polymerase (5 U/μl), 1 μl of each primer (10 μM), 1 μl of DNA template and 18.8 μl sterile water. The cycling conditions were an initial denaturation step at 94 °C for 5 min, followed by 35 cycles of denaturation at 94 °C for 40 s, annealing at 52–54 °C for 40 s and extension at 72 °C for 1 min, with a final extension at 72 °C for 10 min. PCR products were visualised on 1% agarose gels and sequenced using an ABI 3730 DNA Analyzer (Sangon Biotech, Shanghai, China).

### ﻿Sequence alignment and phylogenetic analysis

The newly-generated sequences were assembled and edited using SeqMan (DNASTAR Lasergene 9) and deposited in GenBank (https://www.ncbi.nlm.nih.gov). Sequence alignment was performed using MAFFT v.7.490 (Katoh and Standley 2013) under the default parameters. The resulting alignment was manually adjusted in BioEdit 7.0.9 (Hall 1999) and curated using ClustalX v.1.83 (Thompson et al. 1997; Larkin et al. 2007) to trim ambiguous regions at the ends. The final dataset included 28 newly-generated sequences along with related sequences obtained from GenBank. Phylogenetic trees were visualised using FigTree v.1.4.3 (http://tree.bio.ed.ac.uk/software/figtree/) and graphically refined for presentation using Adobe Illustrator CC 25.4.1. All sequences and relevant information used in the phylogenetic analyses, together with associated references, are provided in Table 1.

**Table 1.** The specimens and GenBank accession numbers for phylogenetic studies. Bold refers to the sequences produced from this study.

Taxon	Voucher	Locality	ITS	nrLSU	References
*Clitocybula abundans*	STU:SMNS-B-FU-2017/00898	Germany	MF627833	-	from GenBank
*Gerronema angustum*	GDGM 88662	China	PQ452698	PQ350413	Zhang et al. 2025
*G. angustum*	GDGM 88663	China	PQ452699	-	Zhang et al. 2025
*G. atrovirens*	BKF10265	Thailand	MZ452668	MZ452672	from GenBank
*G. atrovirens*	BKF10264	Thailand	MZ452088	MZ452671	from GenBank
*G. baishanzuense*	FFAAS0359	China	OL985962	OL985984	Na et al. 2022
*G. brunneosquamulosum*	FFAAS1032	China	OR238884	OR238896	Na et al. 2024
*G. brunneosquamulosum*	FFAAS1033	China	OR238885	OR238897	Na et al. 2024
*G. brunneosquamulosum*	**MHKMU FCR-001**	**Hainan, China**	**PV862907**	**PV862920**	**This study**
*G. brunneosquamulosum*	**MHKMU ZWH-838**	**Guizhou, China**	**PV862908**	**PV862921**	**This study**
*G. brunneosquamulosum*	**MHKMU ZWH-839-1**	**Guangxi, China**	**PV862909**	**PV862922**	**This study**
*G. brunneosquamulosum*	**MHKMU ZWH-840**	**Sichuan, China**	**PV862910**	**PV862923**	**This study**
*G. brunneosquamulosum*	**GZDZ**	**Guizhou, China**	PQ058596	**PV862924**	**Li et al. 2025; This study**
*G. brunneosquamulosum*	**SCHJII**	**Sichuan, China**	PQ058598	**PV862925**	**Li et al. 2025; This study**
*G. brunneosquamulosum*	**SCHJI**	**Sichuan, China**	PQ058599	**PV862926**	**Li et al. 2025; This study**
*G. brunneosquamulosum*	**CQNC**	**Chongqing, China**	PQ058593	**PV862927**	**Li et al. 2025; This study**
*G. brunneosquamulosum*	**CQW**L	**Chongqing, China**	PQ058594	**PV862928**	**Li et al. 2025; This study**
*G. brunneosquamulosum*	**LWBZ**	**Chongqing, China**	PQ058597	**PV862929**	**Li et al. 2025; This study**
*G. brunneosquamulosum*	**MHKMU SLJ-213**	**Anhui, China**	**PV862911**	**PV862930**	**This study**
*G. brunneosquamulosum*	**MHKMU DQ-063**	**Anhui, China**	**PV862912**	**PV862931**	**This study**
*G. brunneosquamulosum*	**MHKMU TLP-3572**	**Yunnan, China**	-	**PV862932**	**This study**
*G. brunneosquamulosum*	**MHKMU TLP-3573**	**Yunnan, China**	-	**PV862933**	**This study**
*G. confusum*	BJTC FM1624	China	OK161271	-	from GenBank
*C. familia*	PRM 921866	Czech Republic	JF730327	JF730320	Antonín et al. 2011
*G. flavum*	BKF10258	Thailand	MZ452142	MZ452170	Zhang et al. 2024b
*G. indigoticum*	HMJAU47636	Guangxi, China	NR166278	MK693732	Liu et al. 2019
*G. indigoticum*	HMJAU47942	Guangxi, China	MK693728	MK693733	Liu et al. 2019
*G. keralense*	CAL1666	India	NR159832	-	Latha et al. 2018
*G. keralense*	BKF10263	Thailand	MZ452107	MZ452144	from GenBank
*G. kuruvense*	CAL1665	India	NR159831	-	Latha et al. 2018
*G. kuruvense*	BKF10266	Thailand	MZ452090	BKF10266	from GenBank
*G. lapidescens*	GDGM85271-2	Guangdong, China	OR736198	-	Zhang et al. 2024b
*G. lapidescens*	GDGM85271-3	Guangdong, China	OR736199	-	Zhang et al. 2024b
*G. microcarpum*	FFAAS0373	Zhejiang, China	OL985970	OL985992	from GenBank
*G. lapidescens*	GDGM85271-3	Guangdong, China	OR736199	-	Zhang et al. 2024b
*G. microcarpum*	FFAAS0373	China	OL985970	OL985992	from GenBank
*G. nemorale*	KACC43600	Korea	EU883593	-	Na et al. 2022
*G. nemorale*	KACC43599	Korea	EU883592	-	Na et al. 2022
*G. pubescence*	GDGM 93936	China	PQ452700	PQ350414	Zhang et al. 2025
*G. pubescence*	GDGM 94001	China	PQ452701	PQ350415	Zhang et al. 2025
*G. sinense*	**MHKMU TLP-3502**	**Yunnan, China**	**PV862913**	**PV862934**	**This study**
*G. sinense*	**CQWS**	**Chongqing, China**	**PV862914**	**PV862935**	**This study**
*G. sinense*	**CQYY**	**Chongqing, China**	**PV862915**	**PV862936**	**This study**
*G. sinense*	**SCWY**	**Sichuan, China**	**PV862916**	**PV862937**	**This study**
*G. sinense*	**MHKMU MM-642**	**Hainan, China**	**PV862917**	**PV862938**	**This study**
*Gerronema* sp.	HMJAU59018	China	OK491123	-	from GenBank
*G. strombodes*	TENN62207	USA	FJ596790	-	Hughes et al. 2009
*G. subclavatum*	FLAS-F-60986	USA	MH016932	-	from GenBank
*G. subclavatum*	Redhead-5175-DAOM	not indicated	U66434	-	Lutzoni 1997
*G. waikanaense*	PDD:87667	New Zealand	JQ694117	-	from GenBank
*G. wildpretii*	BRNM788347	Portugal	LT854045	-	Antonín et al. 2019
*G. xanthophyllum*	PRM924657	Czech Republic	LT854023	-	Antonín et al. 2019
*G. xanthophyllum*	IBL43	Poland	MZ410672	-	from GenBank
*G. zhujian*	FFAAS0376	Fujian, China	OL985975	OL985996	Na et al. 2022
*Hydropus fuliginarius*	DAOM196062	USA	-	AF261368	Moncalvo et al. 2002
*H. marginellus*	OSC 112834	USA	EU669314	EU852808	Direct Submission
*Leucoinocybe danxiashanensis*	GDGM80184	China	MZ667478	MZ667482	Direct Submission
*L. lishuiensis*	FFAAS 0115	China	MW424491	MW424495	Na et al. 2021
*Moniliophthora atlantica*	MN-2022a	-	ON180677	-	from GenBank
*M. aurantiaca*	UTC253824	American Samoa	JN692482	JN692483	Kropp and Albee-Scott 2012
*M. brasiliensis*	DIS396c	-	OR228903	-	from GenBank
*M. canescens*	FHMU3156	China	MW826222	MW826226	from GenBank
*M. capitata*	LAH37907	Pakistan	OR387288	OR387125	Haqnawaz el al. 2024
*M. capitata*	LAH37908	Pakistan	OR387289	OR387126	Haqnawaz el al. 2024
M. conchata var. brevispora	BRNM751596	Republic of Korea	KF380834	KF380838	Antonín et al. 2014
*M. mayarum*	DJLBZ511	Belize	MT162718	MT162714	Niveiro et al. 2020
*M. pakistanica*	LAH36969	Pakistan	MZ766451	OM033490	Izhar et al. 2022
*M. pakistanica*	LAH36957	Pakistan	MZ766452	OM033491	Izhar et al. 2022
*M. perniciosa*	MCA2520	Ecuador	AY916743	AY916742	Aime and Phillips-Mora 2005
*M. purpurensis*	LAH37901	Pakistan	OR387292	OR387122	Haqnawaz el al. 2024
*M. purpurensis*	LAH37902	Pakistan	OR387293	OR387123	Haqnawaz el al. 2024
*M. roreri*	MCA2953	Mexico	DQ222925	DQ222926	Phillips-Mora et al. 2006a
*M. roreri*	MCA2954	Belize	DQ222927	DQ222928	Phillips-Mora et al. 2006b
M. roreri var. roreri	IMI389647	-	AY230254	-	from GenBank
*M. sclerotium*	**MHKMU P28**	**Yunnan, China**	**PV862918**	**PV862940**	**This study**
*M. sclerotium*	**MHKMU L1-1**	**Yunnan, China**	**PV862919**	**PV862939**	**This study**
*Moniliophthora* sp.	MCA2501	United States	MT162719	MT162715	Niveiro et al. 2020
*Moniliophthora* sp.	MCA2500	United States	AY916754	AY916752	Aime and Phillips-Mora 2005
*M. ticoi*	Niveiro2249	Argentina	MT162720	MT162716	Niveiro et al. 2020
*M. ticoi*	NY00511157	Bolivia	MT162721	MT162717	Niveiro et al. 2020
*Paramarasmius mesosporus*	TNSF_48339	Japan	OM522625	OM522623	Vladimír et al. 2023
*P. mesosporus*	BRNM_828732	South Korea	OM522626	OM522619	Vladimír et al. 2023
*P. palmivorus*	MMPS27	Malaysia	MN871733	MN934816	Maizatul-Suriza et al. 2021
*P. palmivorus*	Kirschner4194	Taiwan	MK713652	MN636737	Pham et al. 2020

To reduce topological complexity and increase phylogenetic resolution, two separate phylogenetic trees were constructed for *Gerronema* (Dataset I) and *Moniliophthora* (Dataset II), as they belong to different families. Outgroup taxa for Dataset I were selected according to Zhang et al. (2025), while those for Dataset II were selected according to Oliveira et al. (2024).

The combined ITS-nrLSU dataset was analysed using Maximum Likelihood (ML) and Bayesian Inference (BI). ML analyses with 1,000 rapid bootstrap replicates were performed in RAxML v.7.0.3 (Stamatakis et al. 2008) using the default GTRGAMMA model. BI was conducted on the CIPRES server (Miller et al. 2010) via MrBayes v.3.2 (Ronquist et al. 2012) with four incrementally heated simultaneous Markov Chain Monte Carlo (MCMC) chains run for 1,000,000 generations for the concatenated dataset, sampled every 1,000 generations. The best-fit nucleotide substitution model (GTR+I+G) for both *Gerronema* and *Moniliophthora* was selected using MrModelTest v.2.3 (Nylander 2004), based on the Akaike Information Criterion (AIC). The average deviation of split frequencies was below 0.01 at the end of the run. Burn-in values were determined using Tracer v.1.7 (Rambaut et al. 2018) by examining trace plot convergence and likelihood stabilisation. The first 25% of generations were discarded as burn-in when the sump command-generated plot levelled off and effective sample sizes for all sampled parameters in each run exceeded 200. Bootstrap values (MLB) ≥ 50% and Bayesian posterior probabilities (BPP) ≥ 0.90 were considered significant.

## ﻿Results

### ﻿Phylogenetic analysis

Dataset I included 80 sequences (48 ITS, 32 nrLSU) with a total alignment length of 1692 bp (832 bp ITS, 860 bp nrLSU). Dataset II comprised 80 sequences (26 ITS, 23 nrLSU) with a total alignment length of 1674 bp (790 bp ITS, 884 bp nrLSU). To ensure comprehensive phylogenetic assessment, specimens from various geographic regions were analysed.

**Figure 1.**
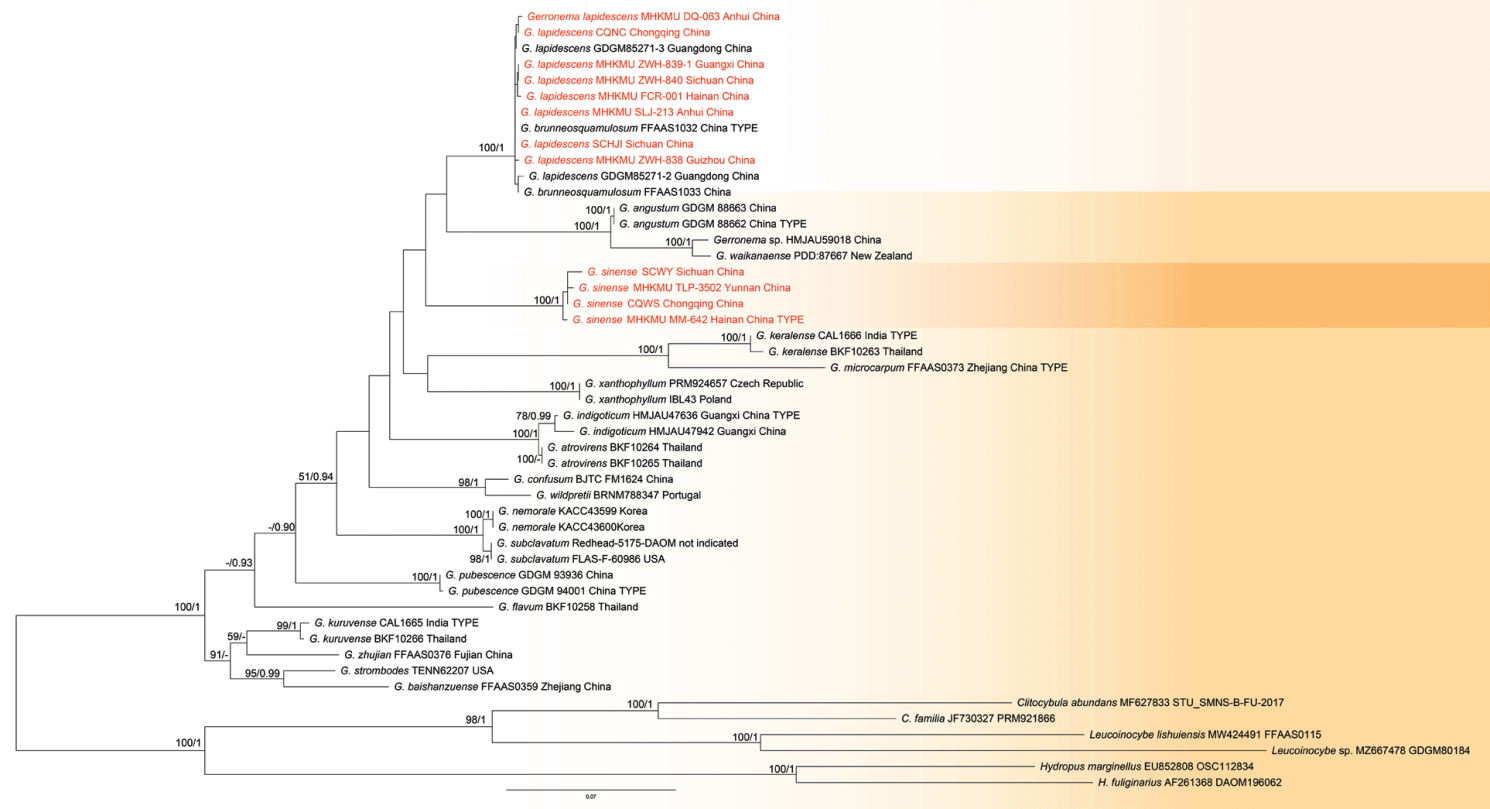
Phylogenetic tree of *Gerronema* inferred, based on ITS-nrLSU dataset. RAxML bootstrap values (MLB ≥ 50%) and Bayesian posterior probabilities (BPP ≥ 0.90) are indicated above the branches. Newly-generated sequences are highlighted in red.

**Figure 2.**
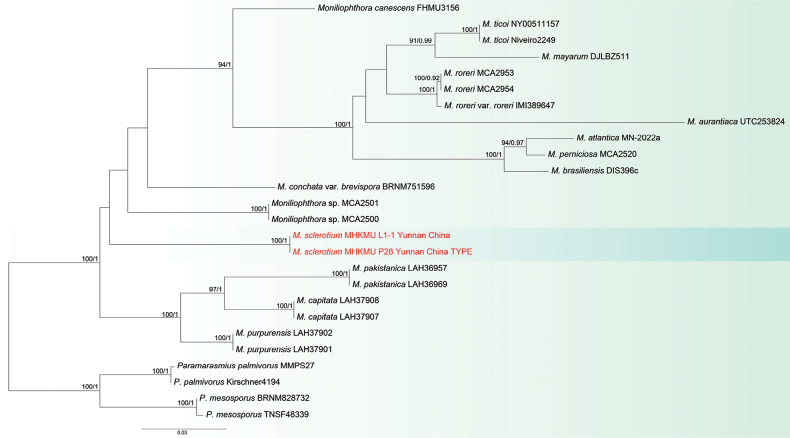
Phylogenetic tree of *Moniliophthora* inferred, based on ITS-nrLSU dataset. RAxML bootstrap values (MLB ≥ 50%) and Bayesian posterior probabilities (BPP ≥ 0.90) are indicated above the branches. Newly-generated sequences are highlighted in red.

The ML and BI analyses of the combined dataset yielded similar topologies (Figs 1, 2). The results indicate that Leiwan specimens are nested within the *Gerronema* and *Moniliophthora* clades. Phylogenetic analysis shows that the specimens of Leiwan represent three distinct species (see Figs 1, 2). Eight specimens (two basidiomata and six sclerotia) clustered with *G. lapidescens* into a single clade. Four specimens (one basidioma and three sclerotia) formed a distinct lineage within *Gerronema*, representing a novel species designated as *G. sinense* sp. nov. Two sclerotial specimens from Yunnan were identified as a new species within *Moniliophthora*, named *M. sclerotium* sp. nov.

### ﻿Taxonomy

#### ﻿ Gerronema brunneosquamulosum

Taxon classificationFungiAgaricalesPorotheleaceae

Q. Na & Y.P. Ge, MycoKeys 105: 49 (2024-04-25)

4AA1C54C-94C5-50FA-943F-8B2FC3B074BB

**Figure 3.**
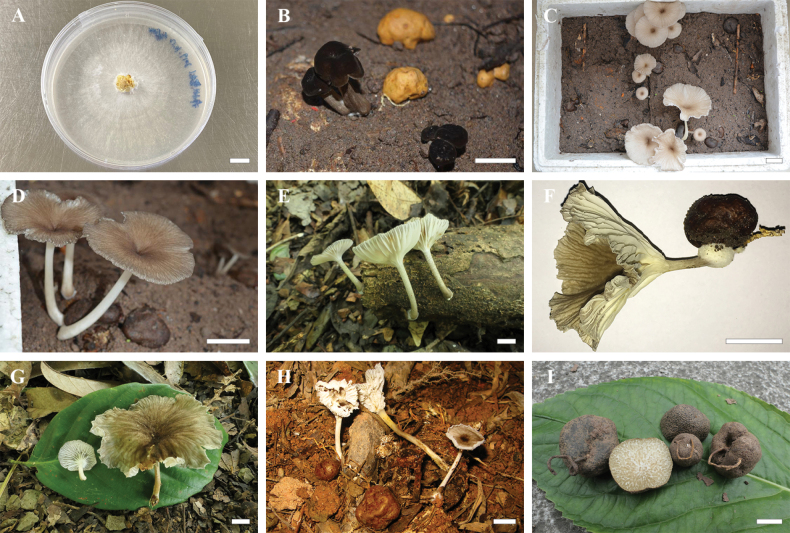
Mycelia, fresh basidiomata and sclerotium of *Gerronema brunneosquamulosum*. **A.** Stage of mycelial development; **B–H.** Fruiting body formation and morphological characteristics of *G. brunneosquamulosum*; **I.** Sclerotium. (**A** from SCHJI, **B** from CQNC; **C** from LWBZ; **D** from LWBZ; **E** from MHKMU DQ-063; **F** from CQNC; **G** from MHKMU SLJ-213, holotype; **H** from CQNC; **I** from MHKMU ZWH-840). Scale bars: 1 cm (**A–I**).

Figs 3, 5, 7D Chinese name: 雷丸老伞

##### Synonym.

*Gerronema lapidescens* (Horan.) Ming Zhang & W.X. Zhang, Acta Edulis Fungi 31: 90 (2024-06-15), syn. nov.

##### Remark.

The following description draws mainly from Na et al. (2024) and Zhang et al. (2024b), supplemented by our field observations and covers macro-morphology, growth habit, geographical distribution and micromorphological characteristics.

##### Description.

***Basidiomata*** small to medium-sized, with a flexible texture. ***Pileus*** 2–6 cm diam., cup- or funnel-shaped, slightly depressed at centre when young, gradually becoming funnel-shaped with growth; initially dark brown (4F5–4F6), becoming progressively lighter with development, transitioning to dark grey-brown (4E5–4E6) and pale grey-brown (2C2) towards the margin; edge paler, greyish-white (2B2), with radial striations or small squamules; surface dry. ***Context*** thin, fragile, yellowish-white (1A2). ***Lamellae*** decurrent, creamy-white, with the same colour on both surfaces and edges, 30–40 pieces of complete lamellae per pileus. ***Stipe*** 1.5–4.5 × 0.1–0.4 cm, central, cylindrical, hollow; surface initially pale white (1A1), developing sparse grey (1B1) scales with age, predominantly scattered along entire stipe; base turning yellow-brown (1B4) at maturity, slightly swollen. ***Odour and taste*** indistinct. The cultured mycelium of *G. brunneosquamulosum* grows prostrately on the surface of the medium, exhibiting a concentric zonation pattern with radially extending hyphae. The colony margin is smooth and well-defined.

***Basidiospores*** [40/2/2] 6.0–8.0 (9.0) × 4.0–5.0 μm (Q = 1.4–2.0, Q_m_ = 1.63 ± 0.2), inamyloid, ellipsoid to elongate-ellipsoid. ***Basidia*** 25–40 × 6–9 μm, clavate, 4-spored, occasionally 2-spored; sterigmata up to 5 μm long. ***Cheilocystidia*** clavate, 18–35 × 4–6 μm and thin-walled. ***Pleurocystidia*** absent. ***Lamellar trama*** subregular; hyphae 2–12.5 μm wide, thin-walled and hyaline. ***Pileipellis*** a cutis; hyphae 2–7 μm wide, light yellow to yellow (1A6–1B6); terminal elements 25–75 × 8–15 μm, clavate, sometimes with sparse coarse excrescences and light yellowish-brown to yellowish-brown (1A5–1B5) pigment in KOH. ***Pileus trama*** subregular, sarcodimitic, sometimes containing dark brown (1C4–1C5) hyphae. ***Stipitipellis*** composed of hyphae 2–10 μm wide, hyaline, smooth. ***Caulocystidia*** 27–55 × 6–12 μm, clavate, thin-walled; light yellowish-brown (2A7–2A5) pigment in KOH. ***Clamp connections*** present in all tissues.

***Sclerotia*** 0.8–3.5 cm diam., elliptical to irregularly globose; peridium thin, brown (2D8–2E8), fissured when fresh, drying dark brown (2E7–2F7) to black, fissures deepened, texture slightly wrinkled. Internal structure dense, firm, hard, often exhibiting white marble-like veins; cut surface showing intertwined wax-yellow (2A2) and milky-white (1A1) striations; comprising irregularly shaped translucent compartments, 1–2 mm wide, separated by white septa; compartments near the outer layer of the sclerotium are typically smaller. ***Odour*** indistinct. ***Rhizomorphs*** brown (2D8) to dark brown (2E7), arising from sclerotia, with vessel hyphae facilitating long-distance translocation of water and nutrients, caducous upon drying. ***Sclerotial context*** primarily composed of compact thick-walled hyphae and viscous substances, hyphae 2–6 μm wide, thick-walled, 1 μm thick, hyaline. ***Clamp connections*** present, rare. ***Sclerotial rind*** with yellowish-brown (2B5–2C5) pigment in KOH.

##### Habit and habitat.

Gregarious on decayed wood in broad-leaved forests dominated by *Cyclobalanopsis glauca* (Thunb.) Oerst. and *Castanopsis eyrei* (Champ.) Tutch. (Fagaceae) or at the bases of bamboo.

##### Distribution.

Known to be primarily in the regions southern of the Yangtze River in China: Anhui, Chongqing, Fujian, Guangdong, Guangxi, Guizhou, Hainan, Sichuan, Yunnan and Zhejiang.

##### Materials examined.

China: Anhui Province: Shitai Prefecture (石台县), Xianyu Town (仙寓镇), Yuantou Village (源头村), 30°3'42"N, 117°17'23"E, elev. 204 m, 1 July 2022, Lin-Jie Su (MHKMU SLJ-213); Shitai Prefecture (石台县), Qidu Town (七都镇), Huanghe Village (黄河村), 30°12'22"N, 117°49'52"E, elev. 284 m, 2 August 2022, Qin Deng (MHKMU DQ-063). Chongqing Province: Nanchuan District (南川区), elev. about 800 m, 8 August 2019, CQNC; Nanchuan District, elev. about 800 m, 2 August 2020, LWBZ; Wulong District (武隆区), elev. about 1,000 m, 2 August 2018, CQWL. Guangxi Province: Duan County (都安县) Longwan Town (龙湾乡), elev. about 400 m, 8 March 2022, Wen-Hao Zhang (MHKMU ZWH-839-1). Guizhou Province: 8 March 2022, Wen-Hao Zhang (MHKMU ZWH-838); Daozhen Prefecture (道真县), elev. 2 August 2020, GZDZ. Hainan Province: 22°51'35"N, 101°1'57"E, elev. 1878 m, 18 September 2020, Cai-Rui Fu (MHKMU FCR-001). Sichuan Province: 5 April 2022, Wen-Hao Zhang (MHKMU WH Zhang 840); Hejiang Prefecture (合江县) elev. 300-500 m, 11 October 2019, SCHJI and SCHJII. Yunnan Province: Lancang Lahu Autonomous County (澜沧拉祜族自治县), 21 July 2021, Li-Ping Tang (MHKMU TLP-3572) and Li-Ping Tang (MHKMU TLP-3573).

##### Notes.

*Gerronema brunneosquamulosum* was originally described by Na et al. (2024) from Zhejiang Province, China and is characterised by a brownish pileus with radial striations and small squamules, elongate basidiospores (Q_m_ = 1.73) and the presence of clamp connections across all tissues. Notably, the original description did not document sclerotial formation. In contrast, *G. lapidescens* was described by Zhang et al. (2024b) from Guangdong Province following isolation from Leiwan sclerotia, with detailed morphological descriptions of the sclerotia provided. In our study, we present a more comprehensive morphological characterisation and demonstrate that all diagnostic features of *G. brunneosquamulosum* align with those of *G. lapidescens*.

Furthermore, phylogenetic analyses, based on ITS and nrLSU sequences, reveal that *G. brunneosquamulosum* and *G. lapidescens* form a highly supported monophyletic clade (see Fig. 1), with negligible genetic divergence — exhibiting only 0–2 bp differences across both the ITS (600 bp) and nrLSU (800 bp) regions. Based on this integrated re-evaluation of morphological and phylogenetic evidence, we conclude that *G. lapidescens* and *G. brunneosquamulosum* are conspecific. In accordance with Article 11 of the International Code of Nomenclature for Algae, Fungi and Plants (ICN; Melbourne Code; McNeill et al. (2012)), which stipulates nomenclatural priority for the earliest validly published name amongst competing synonyms at the species rank, *G. brunneosquamulosum* is hereby recognised as the correct name and *G. lapidescens* is treated as its later synonym. This study supplements the original description by providing comprehensive morphological data for *G. lapidescens* (= *G. brunneosquamulosum*) across all developmental stages, integrating field observations with findings from artificial cultivation.

*Gerronema brunneosquamulosum* is morphologically similar to *G. zhujian* Q. Na, H. Zeng & Y.P. Ge, as both species possess pilei with radial striations or minute scales. However, *G. zhujian* can be distinguished by its smaller basidiomata (0.8–1.8 cm in diameter) and narrower basidiospores (6.7–8.0 × 3.7–4.6 μm).

The comparisons between *G. brunneosquamulosum* and the newly-proposed *G. sinense* are given in the notes for the latter species.

#### ﻿ Gerronema sinense

Taxon classificationFungiAgaricalesPorotheleaceae

L.P. Tang, W.H. Zhang & G.L. Zhang sp. nov.

AA4CE4A6-8290-5850-BAB5-422563BFDDDE

859805

**Figure 4.**
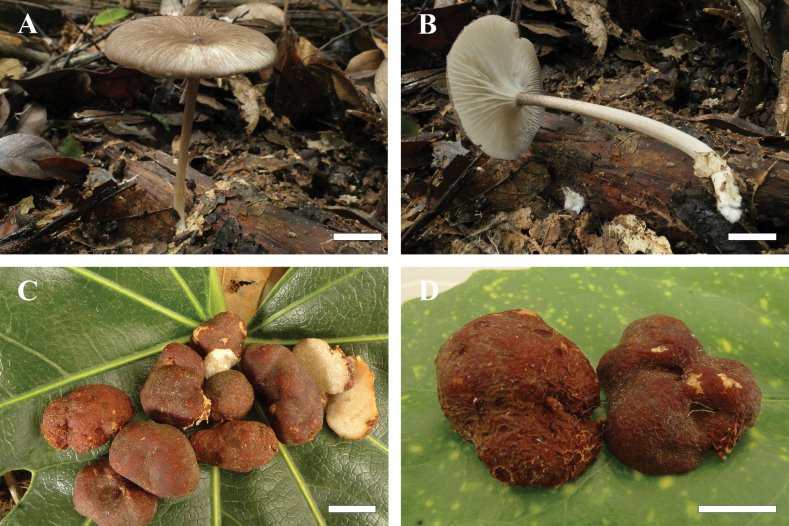
Fresh basidiomata and sclerotium of *Gerronema sinense*. **A, B.***Basidiomata*; **C, D.** Sclerotium (**A, B** from MHKMU MM-642, holotype; **C, D** from MHKMU TLP-3502). Scale bars: 1 cm (**A–D**).

Figs 4, 6, 7E Chinese name: 中华老伞

##### Diagnosis.

Distinguished from other *Gerronema* species by its funnel-shaped pileus with a yellow-brown surface that becomes dark brown at the centre when mature and a pale grey-brown margin with radial striations or small scales. It is also characterised by a slender and hollow stipe covered with grey squamules, ellipsoid to elongate-ellipsoid basidiospores and the presence of clamp connections. Sclerotium primarily composed of compact filamentous hyphae and viscous substances.

##### Etymology.

“*Sinense*” refers to “Chinese”.

##### Holotype.

China: • Hainan Province: Ledong Li Autonomous County (乐东黎族自治县), Jianfengling (尖峰岭), Mingfeng Valley (鸣凤谷), 18°44'16"N, 108°50'11"E, elev. 810 m, 10 August 2020, Man Mu (MHKMU MM-642), GenBank Acc. No.: ITS = PV862917, nrLSU = PV862938.

##### Description.

***Basidiomata*** small and flexible. ***Pileus*** approximately 3.5 cm diam., dry, centrally depressed, umbilicate; surface with prominent radial striations, yellow-brown (2B8–2B6) to darker brown (2D6–2E6) at centre, grey-brown (2C4–2C2) at margin. ***Context*** thin, white (1A1). ***Lamellae*** decurrent, creamy-white (1A1), about 40 pieces of complete lamellae per pileus. ***Stipe*** ca. 6.0 × 0.5 cm, central, cylindrical, hollow; surface densely covered with grey (1B1) scales near the apex, less evident towards the base, which is swollen and paler. ***Odour and taste*** indistinct. The cultured mycelium of *G. sinense* displays slower radial expansion. Colonies are characterised by a dense, floccose to tomentose aerial mycelium that is markedly elevated above the agar surface. The colony edge is irregular and poorly defined.

***Basidiospores*** [40/1/1] (6.5) 7.0–8.5 (9.0) × 4.0–5.0 (6.0) μm [Q = 1.4–1.77 (2.0), Q_m_ = 1.58 ± 0.15], inamyloid, ellipsoid to elongate-ellipsoid. ***Basidia*** 25–35 × 6–8 μm, 4-spored, clavate; sterigmata up to 5 μm long. ***Cheilocystidia*** 18–30 × 2–6 μm, narrowly clavate or subfusiform, thin-walled. ***Pleurocystidia*** absent. ***Lamellar trama*** subregular; hyphae 2–12.5 μm wide, thin-walled, hyaline. ***Pileipellis*** a cutis; hyphae 1.5–8 μm wide, light yellow to yellow (1A6–1B6), occasionally with coarse excrescences; terminal elements 20–120 × 4–12.5 μm, clavate, sometimes with sparse coarse excrescences, light yellowish-brown to yellowish-brown (2B5–2C5) pigment in KOH. ***Pileus trama*** subregular, sarcodimitic, sometimes with yellowish-brown (2B4) to dark brown (2D4) hyphae. ***Stipitipellis*** composed of hyphae, 2–8 μm wide, hyaline, smooth. ***Caulocystidia*** 20–95 × 4–12 μm, cylindrical or clavate, thin-walled; light yellowish-brown (2A7–2A5) pigment in KOH. ***Clamp connections*** present in all tissues.

**Figure 5.**
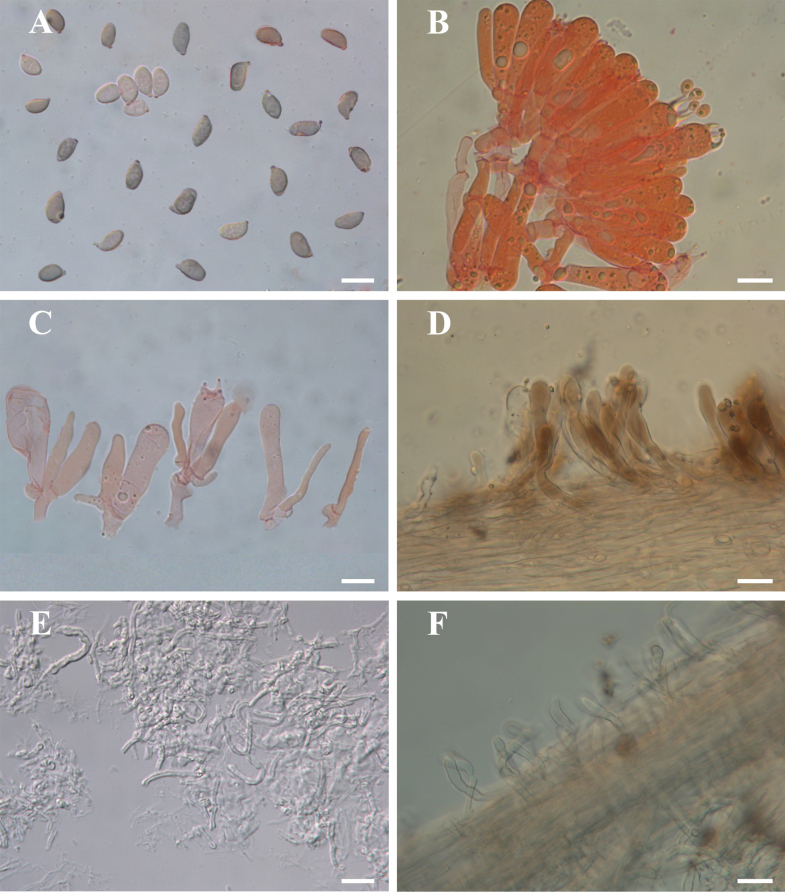
Microscopic features of *Gerronema brunneosquamulosum* (MHKMU SLJ-213). **A.** Basidiospores; **B.** Basidia; **C.** cheilocystidia; **D.** pileipellis; **E.** Sclerotium hyphae; **F.** stipitipellis and caulocystidia. All structures were mounted in 5% KOH. **A–C** were stained with 1% Congo Red. Scale bars: 10 μm (**A–C**); 20 μm (**D–F**).

***Sclerotia*** 1.8–3.5 cm diam., elliptical to irregularly shaped; peridium thin, surface reddish-brown (7C7–7C8) with irregular block-like cracks and fissured when fresh, drying dark purplish-red (11C8–11D8) with deep cracks and slightly wrinkled texture. Internal structure compact and hard, often exhibiting white marble-like veins. ***Odour*** indistinct. ***Rhizomorphs*** reddish-brown (7C7), arising from sclerotia, caducous upon drying. ***Sclerotial context*** primarily composed of compact filamentous hyphae with viscous substances, 1–5 μm wide, thin-walled, hyaline. ***Clamp connections*** present, rare. ***Sclerotial rind*** with yellowish-brown (2B5–2D5) pigment in KOH.

**Figure 6.**
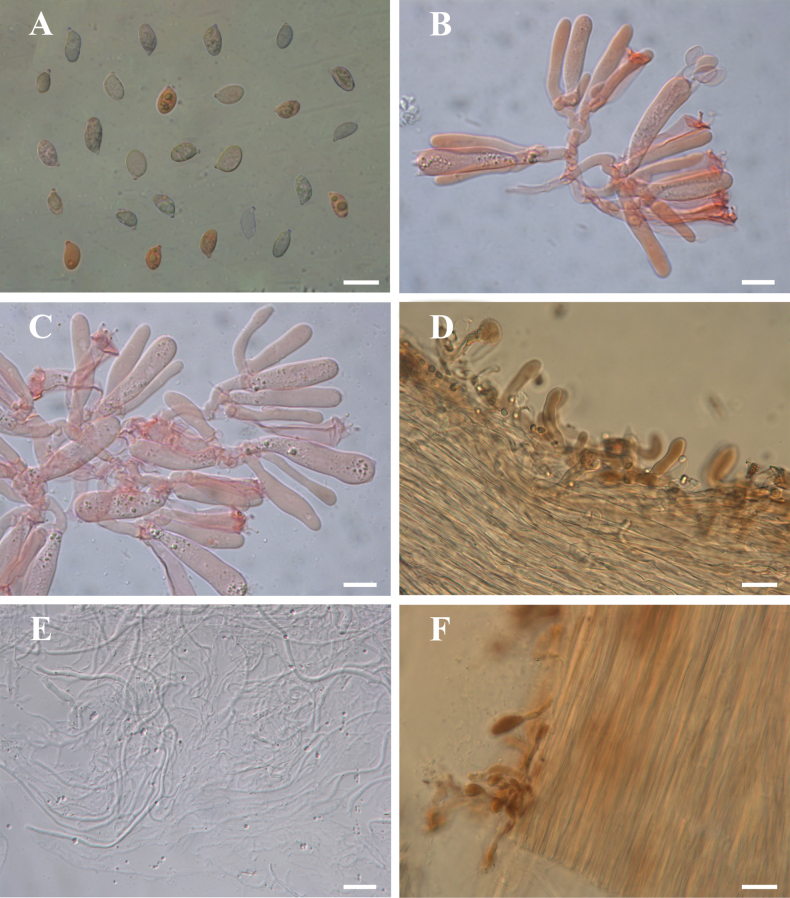
Microscopic features of *Gerronema sinense* (MHKMU MM-642, holotype). **A.** Basidiospores; **B.** Basidia; **C.** cheilocystidia; **D.** Pileipellis; **E.** Sclerotium hyphae; **F.** Stipitipellis and caulocystidia. All structures were mounted in 5% KOH. **A–C** were stained with 1% Congo Red. Scale bars: 10 μm (**A–C**); 20 μm (**D–F**).

##### Habit and habitat.

Scattered to gregarious on decayed wood. Found in broadleaf forests dominated by *Cyclobalanopsis blakei* (Skan) Schottky, *Lithocarpus amygdalifolium* (Skan) Hayata and *Castanopsis carlesii* (Hemsl.) Hayata (Fagaceae).

##### Distribution.

Currently known from south and southwest China: Chongqing, Hainan, Sichuan and Yunnan.

##### Additional materials examined.

China: • Chongqing Province: Wushan County (巫山县), elev. 800–1200 m, 10 August 2019, CQWS; • Yunyang County (云阳县), elev. 400–600 m, 10 August 2018, CQYY. • Sichuan Province: Wanyuan City (万源市), elev. about 900–1200 m, 27 October 2019, SCWY, • Yunnan Province: Puer City (普洱市), Simao District (思茅区), elev. about 1300 m, 18 September 2020, Li-Ping Tang (MHKMU TLP-3502).

##### Notes.

The genus *Gerronema* currently comprises more than eighty species names according to Index Fungorum (http://www.indexfungorum.org, accessed 24 June 2025). Amongst them, only two species, *G. brunneosquamulosum* and *G. sinense*, are known to form sclerotia, which are collectively utilised in traditional Chinese medicine as “Leiwan”. In the phylogenetic tree (Fig. 1), *G. sinense* does not form a well-supported clade with any currently recognised taxa, including *G. brunneosquamulosum*, which supports its status as a distinct species.

Morphologically, *G. brunneosquamulosum* is distinguished by its relatively large basidiomata with the pileus reaching up to 6 cm in diameter. The pileus surface exhibits prominent radial striations at juvenile stages, which become less distinct at maturity. The context is thin and the pileus margin is slightly involute. The stipe is either glabrous or bears sparsely distributed squamules.

Conversely, *G. sinense* produces smaller basidiomata, but retains conspicuous, persistent radial striations throughout its development. The stipe is distinctly adorned with densely aggregated squamules, particularly concentrated in the subapical region. In culture, *G. brunneosquamulosum* exhibits prostrate, radiating mycelia that form concentric rings with smooth margins, whereas *G. sinense* grows more slowly, producing dense, floccose aerial mycelia with irregular colony margins.

A detailed comparison of sclerotial characteristics between these two species and *M. sclerotium* is provided in the notes for the latter.

#### ﻿ Moniliophthora sclerotium

Taxon classificationFungiAgaricalesMarasmiaceae

L.P. Tang, W.H. Zhang & G.L. Zhang sp. nov.

C470C8C8-4065-59FE-8BE8-7DDCA232A216

859806

**Figure 7.**
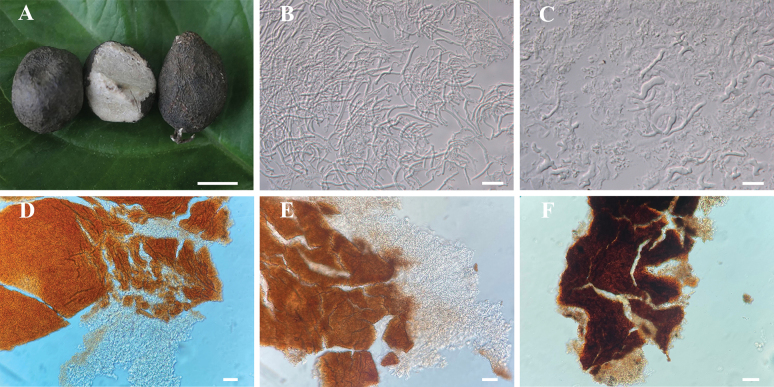
**A–C** Sclerotium and microscopic features of *Moniliophthora sclerotium* (MHKMU P28, holotype). **D–F.** Sclerotial rind (**D.***Gerronema brunneosquamulosum*; **E.***Gerronema sinense*; **F.***Moniliophthora sclerotium*). Structures in **B–F** were mounted in 5% KOH. Scale bars: 1 cm (**A**); 25 μm (**B, C**); 50 μm (**D–F**).

Fig. 7A–C, F Chinese name: 菌核丛梗霉皮伞

##### Diagnosis.

*Sclerotia* relatively small, 0.5–2.5 cm diam., globose to irregularly shaped; outer layer thin, grey-brown to grey-black (2D2–2F2) when fresh, turning dark grey (2F2) when dried. Composed of compact filamentous and vascular hyphae, 1.5–8 μm wide, thin-walled, hyaline. Sclerotial rind yellowish-brown to dark brown pigmentation in KOH.

##### Etymology.

“*sclerotium*” denotes the dense, compact resting structure typical of certain fungi, emphasising its key features.

##### Holotype.

China: • Yunnan Province: 10 August 2020, MHKMU P28, GenBank Acc. No.: ITS = PV862918, nrLSU = PV862940.

##### Description.

***Sclerotia*** globose, elliptical to irregularly shaped, 0.5–2.5 cm diam.; peridium thin, grey-brown to grey-black when fresh, turning dark grey with irregular block-like cracks when dried; internal structure compact and hard, often exhibiting white marble-like veins. ***Odour*** indistinct. ***Sclerotial context*** primarily composed of compact, abundant thin-walled, hyaline hyphae, plus viscous substances; filamentous hyphae 1.5–4 μm wide, vascular hyphae 5–8 μm wide. ***Clamp connections*** present, rare. ***Sclerotial rind*** yellowish-brown to dark brown pigmentation in KOH.

##### Habit and habitat.

Scattered to gregarious on decayed wood.

##### Distribution.

Currently known only from Yunnan Province.

##### Additional materials examined.

China: • Yunnan Province: 10 August 2020, MHKMU L1-1.

##### Notes.

According to Index Fungorum (http://www.indexfungorum.org, accessed 24 June 2025), the genus *Moniliophthora* currently comprises 14 accepted species. Amongst these, *M. sclerotium* is the first and only species reported to form sclerotia, representing a significant expansion of the known ecological and morphological diversity within this genus.

Similar to the sclerotia of *Gerronema* species, those of *M. sclerotium* are also used in traditional Chinese medicine under the name of “Leiwan”. To date, basidiomata of *M. sclerotium* have not been observed; thus, only its sclerotia are described here. Although the sclerotia of *M. sclerotium*, *G. brunneosquamulosum* and *G. sinense* all share a compact internal structure — often exhibiting a marbled pattern in cross-section — they can be reliably distinguished by several morphological features.

Macroscopically, the sclerotia of *M. sclerotium* are comparatively small (0.5–2.5 cm in diameter) and possess a dark outer rind, ranging from grey-brown to grey-black. In contrast, *G. brunneosquamulosum* produces dark brown sclerotia, whereas these of *G. sinense* are distinctly reddish-brown, especially in fresh specimens.

Microscopically, the sclerotia of *M. sclerotium* are characterised by the presence of abundant vascular hyphae, a diagnostic feature not observed in either *Gerronema* species. Furthermore, the sclerotia of *G. brunneosquamulosum* are composed predominantly of dense, thick-walled hyphae, whereas those of *G. sinense* consist mainly of compact filamentous hyphae with less associated viscous material.

By comparison, *L. mylittae*, a species historically suggested as a source of Leiwan (Dai and Yang 2008), differs markedly in sclerotial morphology. Its sclerotia can reach up to 20 cm in diameter and display a distinctive honeycomb-like internal architecture (Robinson 2007), which is inconsistent with the features of authentic Leiwan material derived from the taxa described above.

## ﻿Discussion

Through comprehensive morphological and molecular analyses of specimens collected over twenty years, we have elucidated the taxonomic complexity of Leiwan, demonstrating its multi-origin nature, which encompasses three species from two distinct genera: *G. brunneosquamulosum*, *G. sinense* and *M. sclerotium*. Amongst these, *G. brunneosquamulosum* is the most widely distributed in China, occurring across most provinces south of the Yangtze River Basin and serving as the primary source of medicinal material for Leiwan. *G. sinense* is mainly found in southern and south-western China, while *M. sclerotium* exhibits a narrow ecological range, being restricted to south-western regions.

This study provides compelling molecular and morphological evidence supporting the multi-origin nature of Leiwan. The concurrent discovery and documentation of both basidiomata and sclerotia in *G. brunneosquamulosum* and *G. sinense* offers critical insights into the life history and ecological characteristics of these fungi, establishing a valuable foundation for understanding their natural distribution and development. Morphologically, the sclerotia of *G. brunneosquamulosum* are characterised by thick-walled hyphae, whereas those of *G. sinense* display more compact filamentous hyphae with reduced viscous substances. In contrast, *M. sclerotium*, identified through phylogenetic analysis, is known only from sclerotial specimens. These sclerotia are relatively small and dark and are distinguished microscopically by the presence of abundant vascular hyphae — a feature absent in the *Gerronema* species.

Moreover, comprehensive morphological and phylogenetic analyses, based on two loci, support the treatment of *G. lapidescens* as a late synonym of *G. brunneosquamulosum*, resolving prior taxonomic ambiguities and reinforcing the monophyletic status of this lineage.

From a pharmacognostic perspective, the revelation that Leiwan represents a multi-origin complex has significant implications for its quality control, chemical consistency and pharmacological stability. Previous studies have demonstrated that multi-origin herbal materials can exhibit marked variations in chemical composition and bioactive constituents. For example, distinct *Curcuma* L. species used as Yujin (郁金) and Ezhu (莪术) show notable differences in fatty acid and volatile compound profiles (Zhang et al. 2013). Likewise, chemical profiling of multi-origin traditional Chinese medicines, such as *Ephedrae Herba* and Tinglizi (葶苈子), has revealed pronounced interspecific and batch-level differences in the contents of major active components (Zhang et al. 2024a).

In this context, our demonstration of the polyphyletic nature of Leiwan provides an essential taxonomic framework for subsequent investigations into its secondary metabolites and pharmacological effects. Establishing accurate, source-specific identification criteria will be critical to ensuring the consistency, efficacy and safety of Leiwan-derived medicinal preparations.

## Supplementary Material

XML Treatment for Gerronema brunneosquamulosum

XML Treatment for Gerronema sinense

XML Treatment for Moniliophthora sclerotium

## ﻿Additional information

### Use of AI

No use of AI was reported.

### Author ORCIDs

Wen-Hao Zhang https://orcid.org/0000-0002-8983-7346

Guo-Li Zhang https://orcid.org/0000-0002-9594-0585

Han-Chi Lei https://orcid.org/0009-0009-4805-5354

Chang Xu https://orcid.org/0000-0003-0418-5463

Jia Li https://orcid.org/0009-0007-2442-7008

Xing Xia https://orcid.org/0009-0005-6148-1527

Shuai Jiang https://orcid.org/0000-0003-4852-1940

Na Li https://orcid.org/0009-0009-6493-798X

Li-Ping Tang https://orcid.org/0000-0002-6905-3157

### Data availability

All of the data that support the findings of this study are available in the main text.

## References

[B1] Aime MC Phillips-Mora W (2005) The causal agents of witches’ broom and frosty pod rot of cacao (chocolate, *Theobroma cacao*) form a new lineage of Marasmiaceae. Mycologia 97(5): 1012–1022. 10.3852/mycologia.97.5.1012 16596953

[B2] Antonín V Borovička J Holec J Beran M Dvořák D (2011) *Clitocybula familia* (Fungi, Agaricales) – taxonomy, distribution, ecology and first records in the Czech Republic and Slovakia. Czech Mycology 63(1): 1–11. 10.33585/cmy.63101

[B3] Antonín V Borovička J Holec J Piltaver A Kolařík M (2019) Taxonomic update of *Clitocybula* sensu lato with a new generic classification. Fungal Biology 123(6): 431–447. 10.1016/j.funbio.2019.03.004 31126420

[B4] Antonín V Ryoo R Ka KH Sou HD (2014) Three new species of *Crinipellis* and one new variety of *Moniliophthora* (basidiomycota, marasmiaceae) described from the republic of korea. Phytotaxa 170(2): 86–88. 10.11646/phytotaxa.170.2.2

[B5] Bas C (1969) Morphology and subdivision of *Amanita* and a monograph of its section Lepidella. Persoonia - Molecular Phylogeny and Evolution of Fungi 5(4): 285–573.

[B6] Chen HZ Bao HY (2012) Bentsaological Research of Fungal Medicine *Omphalia lapidescens.* Mycological Research 10(1): 57–62. [In Chinese]

[B7] Chen LC Lu ZX Yang YL Du LJ Zhou XF Chen YT (2018) Effects of purified *Omphalia lapidescens* protein on metastasis, cell cycle, apoptosis, and the JAK-STAT signaling pathway in SGC-7901 human gastric cells. Oncology Letters 15(4): 4161–4170. 10.3892/ol.2018.7800 29541181 PMC5835924

[B8] Dai YC Yang ZL (2008) A revised checklist of medicinal fungi in China. Mycosystema 27(6): 801–824. [In Chinese]

[B9] Evans HC Stalpers JA Samson RA Benny LG (1978) On the taxonomy of *Monilia roreri*, an important pathogen of *Theobroma cacao* in South America. Canadian Journal of Botany 56(20): 2528–2532. 10.1139/b78-305

[B10] Guo W (2014) Overview of the clinical application of Leiwan in ancient and modern times. Liaoning Journal of Traditional Chinese Medicine 41(9): 1866–1867. [In Chinese] 10.13192/j.issn.1000-1719.2014.09.030

[B11] Hall TA (1999) BioEdit: A user-friendly biological sequence alignment editor and analysis program for Windows 95/98/NT. Nucleic Acids Symposium Series 41: 95–98. 10.1021/bk-1999-0734.ch008

[B12] Haqnawaz M Fatima N Niazi AR Khalid AN (2024) Two new species of *Moniliophthora* from Punjab, Pakistan. Phytotaxa 635(2): 137–148. 10.11646/phytotaxa.635.2.3

[B13] Hou XW Peng JS (1952) Anthelmintics. Chinese Medical Journal 5: 391–394. [In Chinese]

[B14] Huang NL (2005) China’s most promising medicinal fungi for development. Shanghai Academy of Agricultural Sciences. [In Chinese]

[B15] Hughes KW Petersen RH Lickey EB (2009) Using heterozygosity to estimate a percentage DNA sequence similarity for environmental species delimitation across basidiomycete fungi. The New Phytologist 182(4): 795–798. 10.1111/j.1469-8137.2009.02802.x 19383108

[B16] Izhar A Asif M Niazi AR Khalid AN (2022) A new crinipelloid species (Marasmiaceae, Agaricales) from Pakistan. Phytotaxa 538(3): 197–212. 10.11646/phytotaxa.538.3.3

[B17] Jin JH (1990) Treatment of 64 cases of intestinal tapeworm disease with Leiwan powder. Inner Mongolia Journal of Traditional Chinese Medicine 9(1): 7–8. [In Chinese]

[B18] Katoh K Standley DM (2013) MAFFT Multiple Sequence Alignment Software Version 7: Improvements in Performance and Usability. Molecular Biology and Evolution 30(4): 772–780. 10.1093/molbev/mst010 23329690 PMC3603318

[B19] Kornerup A Wanscher JH (1981) Taschenlexikon der Farben (3^rd^ edn.). Muster-Schmidt Verlag.

[B20] Kropp BR Albee-Scott S (2012) *Moniliophthora aurantiaca* sp. nov., a Polynesian species occurring in littoral forest. Mycotaxon 120: 493–503. 10.5248/120.493

[B21] Larkin MA Blackshields G Brown NP Chenna R McGettigan PA McWilliam H Valentin F Wallace IM Wilm A Lopez R Thompson JD Gibson TJ Higgins DG (2007) Clustal W and Clustal X version 2.0. Bioinformatics 23(21): 2947–2948. 10.1093/bioinformatics/btm404 17846036

[B22] Latha KPD Nanu S Sharafudheen SA Manimohan P (2018) Two new species of *Gerronema* (Agaricales, Basidiomycota) from Kerala State, India. Phytotaxa 364(1): 1–20. 10.11646/phytotaxa.364.1.5

[B23] Li RZ Chen WR (1957) Report on 20 cases of hookworm disease treated with Leiwan. Shanghai Journal of Traditional Chinese Medicine 5: 22–23. [In Chinese]

[B24] Li N Yang Y Han L Zou Y Chen SY Tan QS (2020) Comparative study on the active components and antioxidant activity of polysaccharides from artificially cultivated and wild Leiwan. Journal of Traditional Chinese Medicine 31(10): 2499–2502. 10.3969/j.issn.1008-0805.2020.10.060 [In Chinese]

[B25] Li N Zhou W Yu ZG Zhao XH Wang H Lu SE Cao WG (2025) Developmental characteristics and domestication of *Gerronema lapidescens.* Mycosystema 44(2): 240202. 10.13346/j.mycosystema.240202 [In Chinese]

[B26] Lian C (1989) Application of Leiwan and Areca in treating 40 cases of pediatric refractory food accumulation and abdominal pain. Hebei Journal of Traditional Chinese Medicine 11(5): 29. [In Chinese]

[B27] Liang RX (2012) Treatment of 35 cases of extensive-stage small cell lung cancer with Leiwan capsules combined with chemotherapy. Journal of Traditional Chinese Medicine 53(9): 782. 10.13288/j.11-2166/r.2012.09.034 [In Chinese]

[B28] Liu YX Lin YL (2006) Colour Atlas of Compendium of Materia Medica. Military Medical Science Press. [In Chinese]

[B29] Liu LN Mou GF Bau T (2019) A new *Gerronema* species with striking colours from China. Phytotaxa 405(2): 74–84. 10.11646/phytotaxa.405.2.2

[B30] Lutzoni F (1997) Phylogeny of lichen- and non-lichen-forming omphalinoid mushrooms and the utility of testing for combinability among multiple data sets. Systematic Biology 46(3): 373–406. 10.1093/sysbio/46.3.373 11975328

[B31] Maizatul-Suriza M Suhanah J Madihah AZ Idris AS Mohidin H (2021) Phylogenetic and pathogenicity evaluation of the marasmioid fungus *Marasmius palmivorus* causing fruit bunch rot disease of oil palm. Forest Pathology 51(1): 12660. 10.1111/efp.12660

[B32] McNeill J Barrie FR Buck WR Demoulin V Greuter W Hawksworth DL Herendeen PS Knapp S Marhold K Prado J Prud’homme van Reine WF Smith GF Wiersema JH (2012) International Code of Nomenclature for algae, fungi, and plants (Melbourne Code) adopted by the Eighteenth International Botanical Congress, Melbourne, Australia, July 2011. Regnum Vegetabile 154. http://www.iapt-taxon.org/nomen/main.php [accessed 19 Jun. 2025]

[B33] Miller MA Pfeiffer W Schwartz T (2010) Creating the CIPRES Science Gateway for inference of large phylogenetic trees. 2010 Gateway Computing Environments Workshop (GCE), 1–8. 10.1109/GCE.2010.5676129

[B34] Moncalvo JM Vilgalys R Redhead SA Johnson JE James TY Aime MC Hofstetter V Verduin SJW Larsson E Baroni TJ Thorn RG Jacobsson S Clémençon H Jr OKM (2002) One hundred and seventeen clades of euagarics. Molecular Phylogenetics and Evolution 23(3): 357–400. 10.1016/S1055-7903(02)00027-1 12099793

[B35] Na Q Hu YP Zeng H Song ZZ Ding H Cheng XH Ge YP (2022) Updated taxonomy on *Gerronema* (Porotheleaceae, Agaricales) with three new taxa and one new record from China. MycoKeys 89: 87–120. 10.3897/mycokeys.89.79864 36760827 PMC9849079

[B36] Na Q Zeng H Hu YP Ding H Ke BR Zeng ZH Liu CJ Cheng XH Ge YP (2024) Morphological and phylogenetic analyses reveal five new species of Porotheleaceae (Agaricales, Basidiomycota) from China. MycoKeys 105: 49–95. 10.3897/mycokeys.105.118826 38708027 PMC11066505

[B37] National Pharmacopoeia Commission (1963) Pharmacopoeia of the People’s Republic of China: 1963 Edition (Part 1). China Medical Science Press. [In Chinese]

[B38] Niveiro N Ramírez NA Michlig A Lodge DJ Aime MC (2020) Studies of Neotropical tree pathogens in *Moniliophthora*: A new species, *M. mayarum*, and new combinations for *Crinipellis ticoi* and *C. brasiliensis*. MycoKeys 66: 39–54. 10.3897/mycokeys.66.48711 PMC7136302 32273793

[B39] Nylander JAA (2004) MrModeltest v2. Program distributed by the author. Uppsala University.

[B40] Oliveira JJS Desjardin DE Jenkinson TS Margaritescu S Capelari M Moncalvo JM (2024) Taxonomic revision of *Marasmius* Fr. and Marasmiaceae Roze ex Kühner based on multigene phylogenetics and morphological evidence. Fungal Diversity 127: 1–54. 10.1007/s13225-024-00534-x

[B41] Pham MT Huang CM Kirschner R (2020) First report of the oil palm disease fungus *Marasmius palmivorus* from Taiwan causing stem rot disease on native Formosa palm *Arenga engleri* as new host. Letters in applied microbiology 70(3): 143–150. 10.1111/lam.13257 31785004

[B42] Phillips-Mora W Cawich J Garnett W Aime MC (2006a) First report of frosty pod rot (moniliasis disease) caused by *Moniliophthora roreri* on cacao in Belize. Plant Pathology 55: 584. 10.1111/j.1365-3059.2006.01378.x

[B43] Phillips-Mora W Coutino A Ortiz CF Lopez AP Hernandez J Aime MC (2006b) First report of *Moniliophthora roreri* causing frosty pod rot (moniliasis disease) of cocoa in Mexico. Plant Pathology 55: 584. 10.1111/j.1365-3059.2006.01418.x

[B44] Preparation Group of National Compendium of Chinese Herbal Medicine (1975) National Compendium of Chinese Herbal Medicine. National Compendium of Chinese Herbal Medicine. [In Chinese]

[B45] Rambaut A Drummond AJ Xie D Baele G Suchard MA (2018) Posterior summarization in Bayesian phylogenetics using Tracer 1.7. Systematic Biology 67(5): 901–904. 10.1093/sysbio/syy032 29718447 PMC6101584

[B46] Robinson R (2007) Fungus of the Month–Laccocephalum mylittae (Native Bread). Science Division, Department of Biodiversity, Conservation and Attractions, Western Australia. https://library.dbca.wa.gov.au/FullTextFiles/PAM03038.pdf [accessed 13 Nov 2025]

[B47] Ronquist F Teslenko M van der Mark P Ayres DL Darling A Höhna S Larget B Liu L Suchard MA Huelsenbeck JP (2012) MrBayes 3.2: Efficient Bayesian phylogenetic inference and model choice across a large model space. Systematic Biology 61(3): 539–542. 10.1093/sysbio/sys029 22357727 PMC3329765

[B48] Singer R (1951) New genera of fungi. V. Mycologia 43(5): 598–604. 10.1080/00275514.1951.12024157

[B49] Stamatakis A Hoover P Rougemont J (2008) A rapid bootstrap algorithm for the RAxML Web servers. Systematic Biology 57(5): 758–771. 10.1080/10635150802429642 18853362

[B50] Tang LP Cai Q Lee SS Buyck B Zhang P Yang ZL (2015) Taxonomy and phylogenetic position of species of Amanita sect. Vaginatae s.l. from tropical Africa. Mycological Progress 14(1): 1–15. 10.1007/s11557-015-1061-z

[B51] Thompson JD Gibson TJ Plewniak F Jeanmougin F Higgins DG (1997) The CLUSTAL_X windows interface: Flexible strategies for multiple sequence alignment aided by quality analysis tools. Nucleic Acids Research 25(24): 4876–4882. 10.1093/nar/25.24.4876 9396791 PMC147148

[B52] Vilgalys R Hester M (1990) Rapid genetic identification and mapping of enzymatically amplified ribosomal DNA from several *Cryptococcus* species. Journal of Bacteriology 172(8): 4238–4246. 10.1128/jb.172.8.4238-4246.1990 2376561 PMC213247

[B53] Vladimír A Kentaro H Miroslav K (2023) Taxonomy and phylogeny of *Paramarasmius* gen. nov. and *Paramarasmius mesosporus*, a worldwide distributed fungus with a strict ecological niche. Plant Biosystem-An International Journal Dealing with all Aspects of Plant Biology 157(2): 286–293. 10.1080/11263504.2022.2100503

[B54] Wang WJ Zhu XY (1989) Anti-inflammatory and immunostimulatory effects of Leiwan polysaccharides. Acta Pharmaceutica Sinica 24(2): 151–154. [In Chinese] 2801139

[B55] Wang H Cheng XH Liu Q Fan FY (2008) Research progress of *Omphalia lapidescens.* Anhui Agricultural Science 36(35): 15526–15527. [In Chinese]

[B56] White TJ Bruns T Lee S Taylor J (1990) Amplification and direct sequencing of fungal ribosomal RNA genes for phylogenies. PCR protocols, a guide to methods and applications 18: 315–322. 10.1016/B978-0-12-372180-8.50042-1

[B57] Yao YH Ma HF (1979) Extraction of Leiwan protease and its preliminary observation on inhibiting mouse sarcoma 180. Journal of Ningxia Medical University 1: 50–52. [In Chinese]

[B58] Yu C (2002) Dictionary of Chinese Veterinary Medicine. Sichuan Science and Technology Press. [In Chinese]

[B59] Zhang GQ Huang YD Bian Y Wong JH Ng TB Wang HX (2006) Hypoglycemic activity of the fungi *Cordyceps militaris*, *Cordyceps sinensis*, *Tricholoma mongolicum*, and *Omphalia lapidescens* in streptozotocin-induced diabetic rats. Applied Microbiology and Biotechnology 72(6): 1152–1156. 10.1007/s00253-006-0411-9 16575562

[B60] Zhang XJ Qiu JF Guo LP Wang Y Li P Yang FQ Su H Wan JB (2013) Discrimination of multi-origin chinese herbal medicines using gas chromatography-mass spectrometry-based fatty acid profiling. Molecules 18(12): 15329–15343. 10.3390/molecules181215329 24335614 PMC6269696

[B61] Zhang Y Wang X Tang F (2024a) Equality Evaluation on a Multi-Sources Traditional Chinese Medicine, Ephedra sinica and Tinglizi. Pharmaceutical Chemistry Journal 58: 1422–1430. 10.1007/s11094-025-03290-7

[B62] Zhang WX Wang CQ Deng WQ Chang CQ Zhang M (2024b) A Taxonomic Revision of Rare Medicinal Fungus Leiwan. Acta Edulis Fungi 31(3). 10.16488/j.cnki.1005-9873.2024.03.010 [In Chinese]

[B63] Zhang WX Deng WQ Chang CQ Zhou P Lin M Zhang M (2025) Three new species of *Gerronema* (Agaricales, Basidiomycota) from southern China. MycoKeys 114: 239–258. 10.3897/mycokeys.114.145299 40079028 PMC11898245

[B64] Zhao GH Xu CB Feng ML You JY Guo MD Li HL (1998) Observation on the histological changes of Taenia solium cysticercus in vitro by Leiwan protease. Chinese Journal of Parasitology and Parasitic Diseases 16(2): 113–116. [In Chinese] 12078218

[B65] Zhu XY Du XM Janson JC Xue LM Li NY Bai JY Cheng GF (2016) Anti-inflammatory and immunomodulatory effects of Leiwan polysaccharide S-4002. Acta Academiae Medicinae Sinicae 38(2): 245–246. 10.3881/j.issn.1000-503X.2016.02.022 [In Chinese]

